# Micro-computed tomography analysis of the lumbar pedicle wall

**DOI:** 10.1371/journal.pone.0253019

**Published:** 2021-07-08

**Authors:** Tomoyo Y. Irie, Tohru Irie, Alejandro A. Espinoza Orías, Kazuyuki Segami, Norimasa Iwasaki, Howard S. An, Nozomu Inoue

**Affiliations:** 1 Department of Orthopedic Surgery, Rush University Medical Center, Chicago, IL, United States of America; 2 Department of Orthopaedic Surgery, Faculty of Medicine and Graduate School of Medicine, Hokkaido University, Sapporo, Japan; University of California San Francisco, UNITED STATES

## Abstract

**Background:**

Although the pedicle is routinely used as a surgical fixation site, the pedicle wall bone area fraction (bone area per unit area) and its distribution at the isthmus of the pedicle remain unknown. The bone area fraction at the pedicle isthmus is an important factor contributing to the strength of pedicle screw constructs. This study investigates the lumbar pedicle wall microstructure based on micro-computed tomography.

**Methods:**

Six fresh-frozen cadaveric lumbar spines were analyzed. Left and right pedicles of each vertebra from L1 to L5 were resected for micro-computed tomography scanning. Data was analyzed with custom-written software to determine regional variation in pedicle wall bone area fraction. The pedicular cross-section was divided into four regions: lateral, medial, cranial, and caudal. The mean bone area fraction values for each region were calculated for all lumbar spine levels.

**Results:**

The lateral region showed lower bone area fraction than the medial region at all spinal levels. Bone area fraction in the medial region was the highest at all levels except for L4, and the median values were 99.8% (95.9–100%). There were significant differences between the lateral region and the caudal region at L1, L2 and L3, but none at L4 and L5. The bone area fraction in the lateral region was less than 64% at all spinal levels and that in the caudal region was less than 67% at the L4 and L5 levels.

**Conclusions:**

This study provides initial detailed data on the lumbar pedicle wall microstructure based on micro-computed tomography. These findings may explain why there is a higher incidence of pedicle screw breach in the pedicle lateral and caudal walls.

## Introduction

Pedicle screw fixation is widely used for stabilization of motion segments of the spine in a variety of spinal disorders. However, pedicular wall penetration and loosening of the pedicle screw can occur especially in osteoporotic subjects [1, 2]. It has been reported that larger pedicle screw diameters provide better cortical purchase of the screw at the isthmus of the pedicle, as it is important in allowing better surgical fixation strength [3–7]. Since cortical bone is denser than trabecular bone, greater insertion torque is required, but it provides enhanced screw purchase [8].

Pedicular wall structural properties have been reported mainly with regards to wall thickness and its cross-sectional histomorphometry [8–14]. Regional variation of the pedicle isthmus thickness and the orientation of the osteons have been previously investigated; the medial wall has been shown to be thicker than the lateral wall [8–12] and the osteons were shown to be colinearly aligned and tightly packed together at the cranial and caudal regions than the medial and the lateral regions [13], respectively. Bone porosity is also an important factor contributing to bone strength [15, 16]. While the majority of the literature regarding bone porosity focuses on the trabecular bone, we only found one study that reported on the distribution of *cortical bone* porosity of the pedicle; percent bone volume/total volume within the cephalad and caudad aspects of the thoracic cortical bone of the pedicle quantified via micro-CT [17]. However, the porosity distribution of the lumbar pedicle isthmus wall, which is an important factor that contributes to the pedicle screw construct strength, has not been analyzed in the literature. Therefore, the purpose of this study was to investigate the pedicle wall porosity, as represented by bone area fraction at the lumbar spine isthmus using micro-CT.

## Materials and methods

### Specimens

Six fresh-frozen lumbar spines (3M/3F, L1-L5) were procured from an accredited tissue bank (Science Care, Phoenix, AZ). The donors had a mean age 60.2±7.2 years old; (range: 50–67 years old); and all were free from spine-related conditions or spine surgery (**Table 1**). The Institutional Review Board at Rush University Medical Center exempted this study because it involves cadaveric human tissue. The specimens were stored at -20°C and were removed from the freezer one day before testing and allowed to thaw slowly at room temperature. The left and right pedicles of each vertebra from L1 to L5 were removed at the pedicle-vertebral body junction and the pedicle-lamina junction maintaining the tissue and ligaments around the pedicles using a low-speed diamond-coated wafer blade (Buehler IsoMet, Buehler, Lake Bluff, IL). A total of sixty lumbar pedicles (L1-L5) were obtained and included in this study.

**Table 1:** Donor demographics.

Donor number	Cause of death	Age (y)	Sex	Weight (kg)	Height (cm)	BMI (kg/m^2^)
1	Chronic obstructive pulmonary disease	54	Female	40.8	162.6	15.4
2	Massive heart attach	50	Female	108.0	160.0	42.2
3	Cardiac arrest	66	Female	65.3	165.1	24.0
4	Atherosclerotic heart disease	67	Male	52.2	175.3	17.0
5	Chronic heart failure	58	Male	127.5	193.0	34.2
6	Cardiac arrest	66	Male	67.6	177.8	21.4

### Micro-CT imaging

The pedicles were scanned using a micro-computed tomography (μCT) scanner (Scanco μCT 50, Scanco GmbH, Brutisellen, Switzerland). Scanning was performed with the specimen immersed in distilled water inside a 35mm inner diameter sample container tube and held by gauze. The tube was closed with paraffin film to minimize evaporation during the scan. The μCT scan settings were 55kV, 145μA, at a resolution of 34.4μm voxel size and a matrix size of 1024×1024. Axial slice raw image DICOM data were imported to a 3D reconstruction software package (Mimics, Materialise Inc., Leuven, Belgium) for visualization in different anatomical planes and re-slicing in three-dimensional arbitrary planes.

### Determination of the pedicle isthmus and four regions

**Fig 1:**
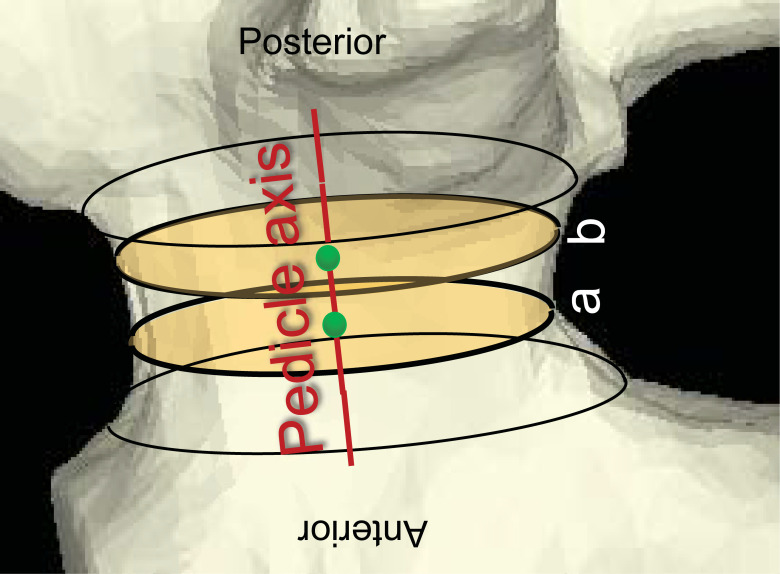
Determination of the pedicle axis at the 3D CT model. The green spheres are centroids at cross sections located at the anterior one-third and the posterior one-third of the pedicle. The yellow ellipses show the cross sections located at the anterior one-third and the posterior one-third of the pedicle. The red line is axis of the pedicle.

**Fig 2:**
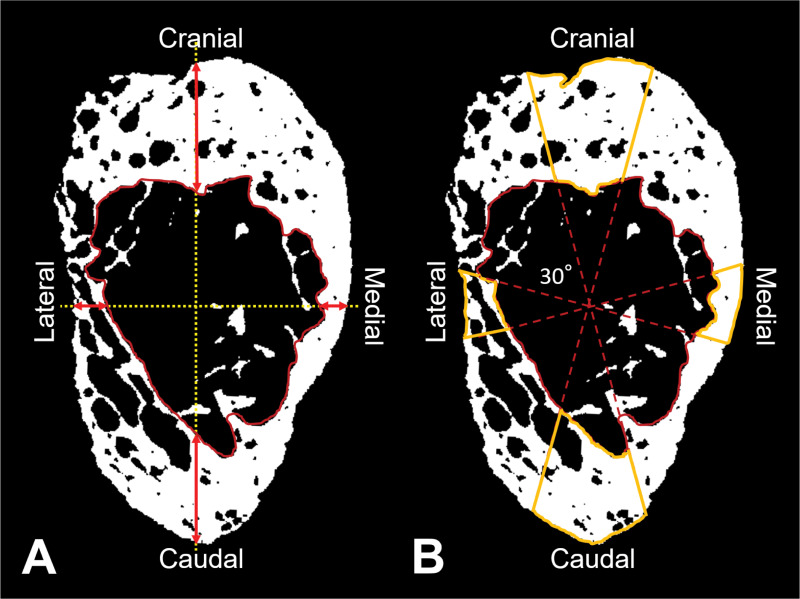
The pedicle dimensions and the four regions (ROIs) for analyses of bone area fraction overlaid on the binarized μCT image. A: The yellow vertical and horizontal dotted lines depict the major and minor axes, respectively. The red arrows show the thicknesses of the medial, lateral, cranial and caudal wall. The area surrounded by the red solid line is the medullary cavity area. B: Orange solid line contours outline the ROIs for measurement of bone area fraction at each region.

The pedicle axis was determined as a linear segment connecting two approximate center points at cross sections located at the anterior one-third and the posterior one-third of the pedicle (**Fig 1**). The algorithm we employed used these points as seed points to find the final cross sections of the pedicle, which were defined as planes perpendicular to the pedicle axis. Since the pedicle cross section can be considered as elliptical, both the minor and major axes were studied in this analysis. The isthmus of the pedicle was defined as the cross-section with the shortest minor axis (in transverse orientation) throughout the pedicle [18]. The major axis of the pedicle isthmus was defined as the longest diameter of the pedicle isthmus. The boundary between the cortical wall and the medullary cavity was defined as a continuous cortical wall adjacent to the medullary cavity referring to a few slices before and after pedicle isthmus [16]. The isthmus was subdivided into four regions, which were determined as follows: a) cranial, b) medial, c) caudal, and d) lateral, and the mean bone area fraction (described later) of each of the four regions (ROIs) was calculated for all spine levels (L1-L5) at this cross-section. The four regions were defined by angular sectors spanning 30° intervals using the cranial direction as the reference (**Fig 2**) with the following ranges: cranial: ±15°; medial: 75°-105°; caudal: 165°-195°; and lateral: 255°-285° from the 12 o’clock position determined by the major axis. The 30˚ ROIs were selected to exclude transitional areas between the four regions.

### Measurement of the pedicle isthmus dimensions

The transverse and longitudinal diameters were defined as the lengths on the minor and major axes of the pedicle isthmus, respectively. The thicknesses of the medial and lateral walls were measured on the minor axis, and the thicknesses of the cranial and caudal walls were measured on the major axis. The areas of entire cross section and medullary cavity were also measured (**Fig 2A**). Each value was measured using ImageJ (US National Institutes of Health, Bethesda, MD).

### Measurement of bone area fraction

Each μCT data set at the isthmus of each pedicle was imported into a custom-written Visual C++ routine that determined pedicle wall bone area fraction (%). The image was binarized using the following equation for threshold level: Threshold level = (cortical bone gray level—background gray level) × 0.5 (50% of offset cortical bone gray level). The bone area fraction at each ROI was calculated as the total pixel number of the bone tissue within each ROI (white pixels in **Fig 2**) divided by the total pixel number within each ROI presented as a percentage (*i*.*e*.: 100%; ROI is filled with all bone tissue, 0%; no bone tissue in the ROI).

### Measurement of attenuation in the vertebral body

Attenuation in the vertebral body was measured using the software ImageJ (US National Institutes of Health, Bethesda, MD) to evaluate osteoporotic changes in the vertebral bodies. For each vertebral body, the largest possible elliptical ROI was drawn for the vertebral body Hounsfield unit (HU) measurement [19]. These ROIs were acquired on all the axial CT slices of the L1–L5 lumbar levels. Osseous abnormalities and voids such as vascular channels were excluded from the ROIs.

### Statistical analyses

Right and left pedicles in the same vertebral level were compared via a paired Student’s *t*-test. Regional and level effects were analyzed using repeated-measures ANOVA with Tukey’s *post hoc* test to probe for statistical differences. Correlations between each bone area fraction and the vertebral body HU values at each spinal level were assessed with Pearson’s correlation coefficients. Results were presented as mean ± SD. Significance was set at p < 0.05.

## Results

The dimensions of the pedicle isthmus from each donor are shown in **Table 2**. The transverse diameter of the isthmus increased gradually from L1 to L5. The longitudinal diameter increased gradually from L2 to L5. The thickness of the lateral wall also showed a gradual increase from L1 to L5. The thickness of the medial wall tended to be higher in lower levels but did not show statistical differences. The cranial and caudal thicknesses did not show a gradual increase in the lower levels. The cranial and caudal wall thicknesses were larger than those of the lateral and medial walls at all levels except L5. No differences were found between the medial wall thickness and the lateral wall thickness at all levels. The areas of entire cross section and medullary cavity at the isthmus gradually increased from L2 to L5.

**Table 2:** Pedicle dimensions.

	L1	L2	L3	L4	L5	Average
Transverse diameter (mm)	7.1 ± 2.4	7.3 ± 2.4	9.4 ± 2.0*	10.6 ± 0.8**	11.8 ± 1.7	9.2 ± 2.1
Longitudinal diameter (mm)	17.0 ± 2.7	15.8 ± 1.7	15.8 ± 1.7	18.8 ± 1.6^†^	24.2 ± 2.7^‡^	18.3 ± 3.5
Medial wall thickness (mm)	0.8 ± 0.2	0.8 ± 0.2	0.8 ± 0.2	1.3 ± 0.5	1.3 ± 0.6	1.0 ± 0.3
Lateral wall thickness (mm)	0.6 ± 0.3	0.7 ± 0.2	0.9 ± 0.4	1.1 ± 0.5	1.7 ± 0.5^§^	1.0 ± 0.5
Cranial wall thickness (mm)	3.1 ± 0.7^a^	3.5 ± 0.9^c^	3.9 ± 0.8^||^^,^ ^e^	3.2 ± 0.8^g^	4.0 ± 1.7	3.5 ± 0.4
Caudal wall thickness (mm)	2.6 ± 0.6^b^	3.2 ± 0.5^d^	2.8 ± 0.9^f^	2.5 ± 1.1	2.9 ± 1.1	2.8 ± 0.3
Whole cross section area (mm^2^)	95.7 ± 43.8	92.5 ± 35.7	113.7 ± 26.4	152.4 ± 13.6^¶^	204.2 ± 37.1^††^	131.7 ± 47.0
Medullary cavity area (mm^2^)	53.2 ± 28.3	43.3 ± 18.4	56.3 ± 19.4	83.2 ± 12.8*	118.7 ± 25.7^‡‡^	70.9 ± 30.5

*: compared with L2 (p<0.04) in transverse diameter and in area of medullary cavity.

**: compared with L1 (p<0.03) and L2 (p<0.05) in transverse diameter.

^†^: compared with L2 (p<0.02) and L3 (p = 0.0001) in longitudinal diameter.

^‡^: compared with L1 (p<0.02), L2 (p<0.003) and L3 (p<0.001) in longitudinal diameter.

^§^: compared with L1 (p<0.03), L2 (p<0.008) and L3 (p<0.05) in thicknesses of lateral wall.

^||^: compared with L1 (p<0.02) in thicknesses of superior wall.

^¶^: compared with L2 (p<0.02) and L3 (p<0.04) in area of entire cross section.

^††^: compared with L1 (p<0.02), L2 (p<0.009) and L3 (p<0.04) in area of entire cross section.

^‡‡^: compared with L1 (p<0.04) and L2 (p<0.02) in area of medullary cavity.

^a^: compared with lateral wall (p<0.007) and medial wall (p<0.003) in L1.

^b^: compared with lateral wall (p<0.005) and medial wall (p<0.002) in L1.

^c^: compared with lateral wall (p<0.004) and medial wall (p<0.003) in L2.

^d^: compared with lateral wall (p<0.0001) and medial wall (p<0.007) in L2.

^e^: compared with lateral wall (p<0.003) and medial wall (p<0.002) in L3.

^f^: compared with lateral wall (p<0.02) and medial wall (p<0.02) in L3.

^g^: compared with lateral wall (p<0.02) and medial wall (p<0.02) in L4.

The bone area fraction of the pedicle wall of each donor is shown in **Table 3.** The bone area fraction values were symmetric between both right and left sides. Since there were no significant differences between them in all levels, the average of the left and right values was used as the result. Two subjects (one was 66 years old male; one was 50 years old female) had vertebral attenuation values at L1 less than 110 HU, which was proposed as a cut-off yielding high specificity for osteoporosis by Pickhardt *et al* [20]. No correlations were found between the average bone area fraction in the pedicle wall and the vertebral body HU values (n = 30, r = 0.06, p = 0.76)

**Table 3:** Bone area fraction (%, mean ± SD) by region.

Donor number	Lateral	Medial	Cranial	Caudal	Average
1	61.7 ± 20.4	99.4 ± 1.2	80.4 ± 15.1	83.2 ± 22.3	81.2 ± 14.7
2	58.9 ± 20.2	95.2 ± 4.4	87.7 ± 9.7	85.5 ± 10.0	81.8 ± 11.1
3	53.2 ± 21.1	93.0 ± 7.6	79.7 ± 10.6	75.1 ± 19.9	75.2 ± 14.8
4	74.3 ± 21.5	99.8 ± 0.7	94.6 ± 6.0	82.6 ± 21.7	87.8 ± 12.5
5	46.4 ± 24.5	98.0 ± 2.9	80.2 ± 14.2	63.7 ± 30.9	72.1 ± 18.1
6	58.0 ± 18.9	99.0 ± 2.7	81.1 ± 8.3	78.3 ± 14.0	79.1 ± 11.0

**Fig 3:**
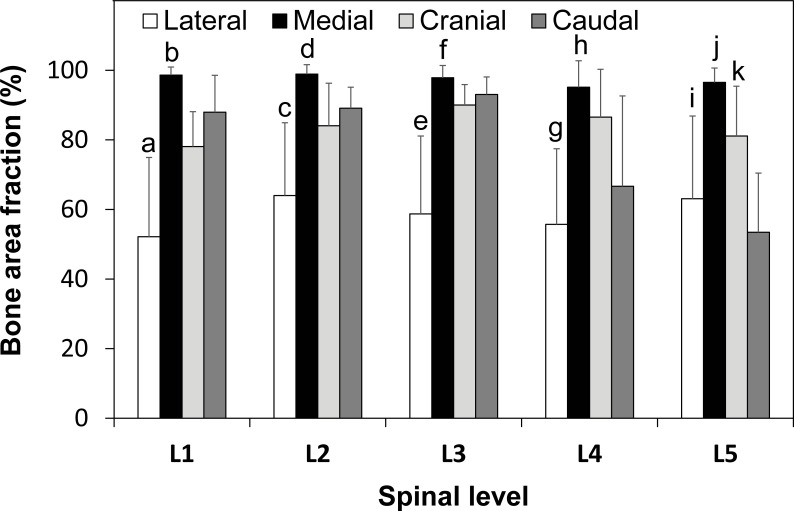
Regional and level comparison of bone area fraction. (mean ± SD). **a**: compared with medial (p<0.0001), cranial (p<0.02) and caudal (p<0.002) in L1. **b**: compared with cranial (p<0.0001) and caudal (p<0.03) in L1. **c**: compared with medial (p<0.0009), cranial (p<0.03) and caudal (p<0.02) in L2. **d**: compared with cranial (p<0.02) and caudal (p<0.0004) in L2. **e**: compared with medial (p<0.0004), cranial (p<0.002) and caudal (p<0.0006) in L3. **f**: compared with cranial (p<0.01) and caudal (p<0.02) in L3. **g**: compared with medial (p<0.0004) and cranial (p<0.002) in L4. **h**: p<0.03 compared caudal in L4. **i**: compared with medial (p<0.002) and cranial (p<0.05) in L5. **j:** compared with cranial (p<0.02) and caudal (p<0.0001) in L5. **k**: compared with caudal (p<0.0006) in L5.

The average bone area fraction of the pedicle wall was 58.8±22.3% in lateral, 97.4±4.1% in medial, 84.0±11.2% in cranial and 78.0±12.9% in caudal regions, respectively (**Table 4**). The bone area fraction in the lateral region was lowest among the four regions at L1, L2 and L3 and significantly lower than that in the medial region at L4 and L5 (p<0.0004 and p<0.002, respectively). The bone area fraction in the medial region was highest among the four regions at all levels except for L4 (p<0.004 for medial vs. lateral, p<0.03 for medial vs. caudal, and p = 0.26 for medial vs. cranial at L4, respectively). The lateral and caudal regions at L1, L2 and L3 showed significant differences between them (p<0.002, p<0.02, and p<0.0006, respectively), but there were no significant differences at L4 and L5. The area fraction at the caudal region was lower than that in the cranial region at L5 (p<0.0006). While the value of the bone area fraction in the medial region was over 95% at all spinal levels, that in the lateral region was less than 64% at all spinal levels and that in the caudal region was less than 67% at L4 and L5 (**Fig 3**, **Table 4**).

**Table 4:** Bone area fraction by spinal level (%, mean ± SD).

Level	Lateral	Medial	Cranial	Caudal	Average
L1	52.2 ± 22.7	98.6 ± 2.3	78.1 ± 10.0*	88.0 ± 10.6	79.2 ± 11.4
L2	64.0 ± 20.9	98.9 ± 2.7	84.1 ± 12.2	89.1 ± 6.0	84.0 ± 10.5
L3	58.7 ± 22.4	97.9 ± 3.5	90.0 ± 5.9	93.0 ± 5.0	84.9 ± 9.2
L4	55.7 ± 21.7	95.1 ± 7.6	86.6 ± 13.7	66.7 ± 26.0**	76.0 ± 17.2
L5	63.1 ± 23.8	96.5 ± 4.1	81.1 ± 14.3	53.4 ± 17.0^†^	73.5 ± 14.8^‡^

*: compared with L3 (p<0.04) in cranial.

**: compared with L3 (p<0.04) in caudal.

^†^: compared with L1 (p<0.04), L2 (p<0.0001) and L3 (p<0.0001) in caudal.

^‡^: compared with L2 (p<0.04) and L3 (p<0.02) in average.

Comparing between different levels within the same region, the cranial area fraction in L1 was lower than in L3 (p<0.04); the caudal L4 area fraction was lower than caudal L3 (p<0.04) and the caudal L5 area fraction was lower than at L1 (p<0.002), L2 (p<0.0001), and L3 (p<0.0001) respectively (**Table 4**).

## Discussion

The present study is the first to evaluate the regional bone area fraction distribution at the isthmus of the lumbar pedicle wall at all lumbar levels using μCT data. Our regional analysis of the bone area fraction distribution showed that the lateral region achieved lowest values at L1, L2 and L3. Shell-like or cancellous bone-like structure, rather than the cortical structure was dominant in the lateral aspect of the pedicle isthmus at all spine levels. Lateral wall breaches are the most common pedicle-screw related failures in thoracolumbar indications [8–10, 12, 21]. A malpositioned screw due to lateral wall breach may be removed, redirected and inserted along the correct axis intraoperatively. However, Goda *et al*. reported that the mean pullout strength for the redirected screws following lateral wall breach was 24.0% less than in the case of correctly aligned screws [22]. Previous studies in the thoracolumbar pedicle found that the lateral wall thickness was thinner than the medial region [8–10, 13, 21], which has been thought to be a reason for the higher rates of incidence of the pedicle penetration seen in the lateral pedicle wall. On the other hand, it has been reported that bone strength decreases as the mineralized bone matrix volume decreases [23]. Lehman *et al*. investigated the differences in bony architecture of the thoracic pedicle using μCT and compared bone density quantified by μCT with biomechanical strength under cantilever bending in cephalad-caudad directions. The authors found that the caudad half of the pedicle had higher percent bone volume/total tissue volume of the cortical bone and withstood higher forces compared with the cephalad aspect. This study indicates the pedicle wall with higher density is a contributing factor for higher mechanical strength. Although multiple factors would be involved in the higher incidence of pedicle screw breach in the lateral side, lower bone density presented as lower bone area fraction in the lateral pedicle wall and cancellous bone-like structure shown in the present study should be considered to be a contributing factor in addition to the thin wall thickness.

In addition to the bone area fraction of the pedicle isthmus wall, the present study measured other pedicle parameters reported in the literature. The studies measuring the lumbar pedicle isthmus dimensions using CT showed increased transverse and longitudinal diameters at lower levels [8, 11, 18], which is consistent with the results in the present study. Limited study has investigated the lumbar pedicle wall thickness using cadaveric specimens by means of direct measurement. Inceoğlu *et al*. reported that the thicknesses of the lateral and medial cortices in the midportion of the cross-section at L3 were 609±247μm (0.609±0.247mm) and 793±186μm (0.793±0.186mm), respectively [13]. The pedicle cortical thickness has been also measured by CT. The cortical thicknesses reported by Mitra *et al*. at L1, L2, L3, L4 and L5 were 0.82, 0.55, 0.87, 0.60 and 1.25mm, respectively, in the lateral wall and 1.07, 1.15, 1.15, 1.37 and 1.32mm, respectively, in the medial wall [11]. Li *et al*. showed the medial wall thickness ranged from 1.4 to 2.3mm in females and 1.5–2.2mm in males; while the lateral wall thickness ranged 0.8–1.6mm in females and 1.0–1.8mm in males with a gradual increase from L1 to L5 [8]. The medial and lateral wall thicknesses measured in the present study were comparable with those measured by direct and CT measurements (**Table 2**).

The bone area fraction in the medial region was highest at all levels except for L4, which showed over 95% bone area fraction in all lumbar cases. Bone area fraction and void fraction (*i*.*e*. porosity) are commonly used parameters to evaluate bone quality. While porosity is often used for the cortical bone, bone area fraction is often used for the cancellous bone. In the present study, bone area fraction was used because some regions of the pedicle, especially the lateral region, showed a cancellous bone-like structure although typical cortical structure was observed in the medial region. The porosity of long bones has been reported by several investigators. Burghardt *et al*. reported that intracortical porosity was 0.3–7.1% (*i*.*e*. 99.7–92.9% in bone area fraction) in the tibia and 0.2–2.4% (*i*.*e*. 99.8–97.6% in bone area fraction) in the radius [24]. Tsai *et al*. also reported that the mean value of cortical porosity was 8.8% (*i*.*e*. 92.2% in bone area fraction) in the tibia and 2.7% (*i*.*e*. 97.3% in bone area fraction) in the radius [25]. The medial region of the pedicle isthmus only showed the bone area fraction in the range seen in typical long bones.

Structural differences between the lateral and medial lumbar pedicle wall have been reported in the literature. Inceoğlu *et al*. histologically reported that the medial wall was significantly thicker than the lateral wall. The authors postulated that the structural difference between the medial and the lateral wall was due to anatomical differences that the lateral aspect of the pedicle connects to the transverse process, whereas the medial aspect of the pedicle connect to the lamina, which causes different loading characteristics between them [13]. These loading distribution and adjacent anatomical structure may also affect decreased bone area fraction in the lateral aspect of the pedicle shown in the present study.

The present study also showed low bone area fraction in the caudal region at L4 and L5. Assuming pedicle cross-sectional geometry has an elliptical shape, the increases in the longitudinal axis at L4 and L5 shown in the present study would increase area moment of inertia to resist a bending moment. While the cranial wall of the pedicle contributes stress transmission to the superior endplate, the reduced bone area fraction in the caudal wall at L4 and L5 may be of limited influence on pedicular structural properties, leading to consider that a reduction of bone area fraction in the caudal wall at L4 and L5 may be a result of functional adaptation of bone tissue under reduced stress caused by the increased area moment of inertia. Looking at higher lumbar levels, however, the bone area fraction of the caudal region tended to be higher than that in the cranial region at L1 (p = 0.08) and significantly higher (p < 0.04) in the combined L1 and L2 levels, which agree with the results of the thoracic study by Lehman *et al*. showing higher percent bone volume/total volume in the caudad half of the thoracic pedicle as compared with cephalad half [17]. Future biomechanical studies on the stress/strain distribution in the pedicle and their correlation with the bone area fraction distribution will be required to explain the regional and spinal level differences in pedicle wall the bone area fraction.

Our results showed no correlation between the area fraction of the pedicle wall and HU values of the cancellous bone in the lumbar vertebral body. This finding may support the concept that progression of osteoporosis at the pedicle wall is independent from that in the cancellous bone rich lumbar vertebral body, which is one of the theoretical justifications for cortical bone trajectory screw fixation [26]. However, the sample size in the present study is limited and therefore future studies will be required to investigate if the porosity of the pedicle wall is independent from BMD in the vertebral body using larger sample size.

**Fig 4:**
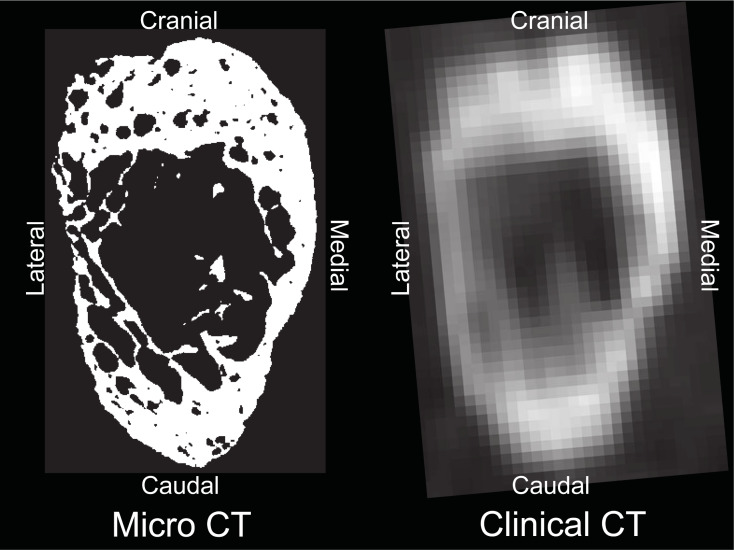
Representative comparison between this study’s μCT scan and clinical CT scan at the pedicular isthmus for the same specimen. scanned Clinical CT image acquisition parameters: Volume Zoom scanner (Siemens, Malvern, PA), tube voltage: 120 kV, tube current: 100 mA, field of view: 272 mm, image matrix: 512×512, slice increment: 1.0 mm, slice thickness: 0.625 mm, no spacing. The difference in resolution is self-evident, leading to less accurate pedicle wall characterization with clinical CT.

Our study has some limitations. First, this study only evaluated the isthmus of the lumbar pedicle. Although the analysis of the pedicle isthmus is relevant for traditional pedicle screw fixation, analyses of other portions of the pedicle would be also important for new fixation techniques such as cortical bone trajectory screw fixation. Future studies will analyze distribution of the bone area fraction through the pedicle length. Second, the small sample size prevented us from establishing high-trustworthiness correlations between the pedicle wall porosity and vertebral body HU values. A larger sample size will be required to adequately power future studies. Thirdly, μCT scanning of the lumbar pedicle is not feasible in a clinical setting. While individual pedicle trabeculae, even within the pedicle wall, could be visualized by μCT scanning with the 34.4μm (0.0344 mm) voxel resolution achieved in the present study, the trabecular structure cannot be visualized by currently available clinical high-resolution CT, which achieves about 0.5 mm voxel resolution at best (**Fig 4**). However, higher porosity of the pedicle wall may be represented by reduction of the CT values due to partial volume effects. Therefore, future studies comparing between the μCT and clinical CT images will be necessary so that the findings of the present study can become clinically relevant and of a translational nature. Lastly, in the present study, ROIs for the measurement of the area fraction were set in the limited areas at the cranial, medial, caudal and lateral regions with angular sectors spanning arbitrarily selected 30˚ intervals aiming to measure the most typical values for each quadrant of the pedicle. Therefore, the area fraction of pedicle wall measured in the present study does not cover all areas of the pedicle wall and area fractions at the transitional areas between the ROIs are not clarified. The complete distribution of the area fraction through the pedicle wall warrants future studies.

In summary, this study provided initial detailed data on the microstructure of the human lumbar spine pedicle isthmus. The results of the present study demonstrated low bone area fractions with values of less than 67% in the lateral region at all spinal levels and in the caudal region at L4 and L5 levels, while only the medial region showed bone area fraction values of over 95% at all spine levels, which are typically seen in the cortex of long bones. The thin lateral wall of the pedicle has been considered as a contributing factor for pedicle screw breach at the lateral wall. The present study demonstrated high bone porosity within a thin lateral wall. The reduced bone quality presented by the high porosity at the lateral wall should be considered as another cause of screw breach and pedicle failure in addition to just thin thickness of the lateral wall.
